# Hume’s guillotine and intelligent technologies

**DOI:** 10.1007/s42454-021-00035-1

**Published:** 2021-08-23

**Authors:** Pertti Saariluoma

**Affiliations:** Information Technology, University of Jyväskylä, 40014 Jyväskylä, Finland

**Keywords:** Hume’s guillotine, Weak and strong ethical AI, Processing ethical information

## Abstract

Emerging intelligent society shall change the way people are organised around their work and consequently also as a society. One approach to investigating intelligent systems and their social influence is information processing. Intelligence is information processing. However, factual and ethical information are different. Facts concern true vs. false, while ethics is about *what should be done*. David Hume recognised a fundamental problem in this respect, which is that facts can be used to derive values. His answer was negative, which is critical for developing intelligent ethical technologies. Hume’s problem is not crucial when values can be assigned to technologies, i.e. weak ethical artificial intelligence (AI), but it is hard when we speak of strong ethical AI, which should generate values from facts. However, this paper argues that Hume’s aporia is grounded on a mistaken juxtaposition of emotions and cognition. In the human mind, all experiences are based on the cooperation of emotions and cognitions. Therefore, Hume’s guillotine is not a real obstacle, but it is possible to use stronger forms of ethical AI to develop new ethics for intelligent society.

## Introduction

The new technological reality is at the doorway of human social life. Many qualitatively new kinds of technologies are emerging. Perhaps the most revolutionary aspect of emerging technologies is characterised by intelligence. These new kinds of technologies are able to perform actions that require intelligence from people. Therefore, new technologies can be used to automate or autotomize complex work processes. These new technologies will thus profoundly change people’s work processes and social lives (Ford 2015, Gungel 2012; Fukuda, 2020; Tegmark 2017).

Intelligent robots, data-analysing information systems and many rule-based machines can perform tasks that have traditionally been done by people, because they require intelligence (Brigsfjord & Govindarajulu 2020, Dignum 2019, Gungel 2012, Mueller, 2020). In industry, the finance sector, social administration, military, aviation, traffic and medicine, for example, intelligent technologies are able to perform a large share of the necessary tasks (Ford 2014,, Fukuda, 2020; Gungel 2012; Tegmark 2017, Yang et al., 2018).

A decade ago, in order to get a new taxation document in Finland one needed to go to the tax office, queue and wait for it for some time. Today it is possible to input a number and get the document back in less than a second. The content and legal form of the process has remained practically the same, but moving the decision-making process from people to intelligent technologies has saved a lot of time. Consequently, tax offices can operate with a much smaller work force. When similar changes are simultaneously emerging all over society, the way people live will change.

Technology has a long history of causing social changes. Each new innovation adopted in society has changed the way people reach their goals (Bernal 1969; Headrick 2009). Technological revolutions such as fire, sailing, navigation, cannons, printing, clocks, steam engines, electricity and nuclear energy have led to new forms of work and social organisation (Bernal 1969).

Most technology-induced social changes have been incremental or limited, but some have been very extensive. For example, steam energy made it possible to organise industries. Industrialisation led to a period of massive (and ongoing) urbanisation as people moved to cities in search of work. This process triggered changes in social structures and morals as modern urban life replaced the old nobility-based social life (Bryson 2020, Dignum 2019, Muller 2020, Leikas et al., 2019).

However, some historical social transformations have been problematic. New systems of working and production have necessarily led to new kinds of social structures, though solving the problems of necessary social transformations has not been easy. These changes often take a considerable amount of time, in order to engage in the necessary social restructuring and develop new ways of thinking that are accepted by society. Ideas such as freedom of thinking, speech, marriage and entrepreneurship became the focus of social development and were established in advanced societies, but this was not an easy process. Some innovative people went to the USA and established the foundations of a new kind of society. In Britain the transition was gradual, but in France the outcome was a period of terror (Schön 2013).

Moreover, human understanding and adoption of the increased capacities of new technologies has been far from smooth. Military experts and leaders during WWI repeatedly sent troops to storm the killing zones with no other outcome but a huge number of casualties. Obviously, generals at the time did not have a clear understanding of the capacity of machine guns and artillery against storming troops (Horne 1962). History is full of examples of the difficulties of adopting large-scale social innovations. There is no reason to think it would be easier in a combined revolution of ICT and machine intelligence. The only good thing is that societies now have time to think and search for good solutions before they lose their grip on the way things go forward.

Since human social actions are organised by ethical rules (which often become law), it makes sense to consider how ethics can guide AI-transformation processes (Bryson 2020; Dignum 2019). People will be replaced by machines; they will lose their jobs and be forced to learn new and difficult things. Different forms of many other future negative attitudes are likely to receive support in the social information sphere. Thus, it is essential to find good ways for transition processes to minimise social problems. A part of the process involves understanding what ethical technologies are like. Can any technology be ethical? Technologies are just electro-physical processes, and it is hard to understand the ethical aspect of the flow of electricity in a wire.

## Information and representations

The core of modern intelligent technologies is their capacity to process information and to let information processes control their operations. Information is information not matter or energy (Wiener 1948, see page 132). Consequently, an important question is: What is information, and how can it serve as a platform for intelligence? The answer to this question could help us understand intelligence. However, the answer is not easy, as it is possible to define the concept of information in many different ways and to consider it from several standpoints.

The most important conceptions have been analytically presented in Floridi (2011, see p. 31 ff.). Some concepts of information arise from mathematical notions, as communication engineers needed to get a measure of information (Kåhre 2002; Shannon 1948; Shannon and Weaver 1949; Wiener 1948). These conceptions are based on the idea that information can be seen as sets of signs. However, in these theories, signs have no specific content, and thus they cannot be used to investigate what information represents. They can only be used to calculate the number of signs and how probable one sign is in sets of signs.

One logical consequence was to pay attention to the semantics of information. The idea was to consider how it was possible to have references for signs. One could call this basic problem of representation symbol grounding (Harnad 1990, Searle 1992). The problem can be studied in linguistic semantics or semiotics, or in concepts of psychological, philosophical, or cognitive scientific concepts (Chandler 2002, Lyons 1977, 1995, Nierenburgh and Raskin 2004).

The main problem associated with grounding symbols is to build a reference from the sign to the referred object, event, or idea. Thus meaning is a relationship between a sign and its reference (Chandler 2002, Saussure1919/1974). The symbol ‘µ’ means horse, if its reference is a horse as a biological creature, dead body, or conception. µ stands for horse in a representation. This kind of semantics can be called sign semantics (Saariluoma 2000). Typical examples of such semantics are logical semantics (Hintikka and Sandu 1997) or behaviourist semantics (Skinner 1957). They define meanings by ‘pointing’ the reference of a symbol.

However, the process of building meanings for signs need not be thought of as assigning meanings to symbols, which is the approach taken in sign semantics. One must also pay attention to the way assignments can be founded. The problems in straightforward pointing were presumably first noticed in formal philosophical frameworks by Ludwig Wittgenstein (1953). One could think that pointing a brick gives meaning to a brick, but how can one point infinity, eternity, electron, possible or allowed? How can one point any abstract and general notions such as redness or car likeness? On can point at red objects, but is a red object the same as the idea of redness? One could say that one does not wish to have numerous examples of good, but an answer to the problem of what good is.

Consequently, it seems necessary to give up the idea of directly ‘pointed’ references between signs and references and to include cognitive emotional and other mental processes. Like Wittgenstein, one could say that the meaning of a word is in its use. However, the use of signs in different kinds of actions is defined in the human mind. Here, one could get help from the classic tripartite semantics of Ogden and Richards (1923, Saariluoma 2000). Signs are assigned meanings through mental concepts or representations in one’s mind. Symbol µ referring to good means what is represented as good in the human mind.

The latter type of semantics could be called cognitive or mental semantics (Saariluoma 2000, Saariluoma and Rousi, 2015). Thus, the meaning of a sign is how its reference is represented in the human mind. There simply is no information unless the sign and reference are connected by mental representations. An argument for mental semantics is the ‘world end thought experiment’ (Saariluoma 2012). The idea of this thought experiment is simple. Let’s assume that (to be timely) a super coronavirus kills all the people. The same effect could also be achieved by nuclear war or serious disturbances in the systems of the upper air layers. Afterwards, all information would still be there, but there would be no human beings to interpret the signs. A century after the end of the human world, an ant could walk over the surface of a famous painting in a gallery or its guidebook, but what information could it process? Nothing that is meaningful for an educated person today. Without an interpreter, there would be no information. This is why one should relate signs to their references through human minds.

Representations thus form the first necessary component of intelligent information processing. An intelligent system must have the capacity to represent. Representing means that there are signs that have references, which can have information content by means of being associated with human mental representations. The information content of signs is the information content of mental representations or their mental content.

## Selective information processing

Intelligent systems are not mere representations. They are also able to process information. Without such a processing capacity they would be static stories of pictures like books or paintings. Intelligent systems can manipulate representations and thus generate representations in imaginable states (Newell and Simon 1972). They follow the given computational rules to be able to foresee possible courses of action. A chess-playing computer, for example, generates webs of possible courses of the game to find the best moves.

The beginning of computational systems was Turing’s work on his ideal computational machine in the mid-1930s (Petzold 2008, Turing, 1937-7). His machine had a tape of 0 and 1’s or representation and a system that could manipulate the signs accordingly to create rules. The machine was a model of a mathematician who calculated on squared paper. The Turing machine could compute all the computable calculations (Petzold 2008). After the war Turing (1950) pointed out that computing machines could process information like people. In some sense, these machines could be used as models of thinking people. The numbers in representations could be seen as Chinese characters or representations of any states of affairs and thus machines could manipulate the symbols. Mathematics as well as symbol manipulation are both tasks that demand intelligence. The transformation rules need not be elementary mathematical operations. They can be chess moves or steering movements of a boat’s rudder (Saariluoma 1995). Thus, one can use computational machines to imitate human thinking to some degree.

Very soon after Turing’s tragic death, a group of American researchers began to think about how machines could be used in tasks requiring intelligent information processes. For example, Herbert Simon and colleagues (Newell et al., 1958; Newell and Simon 1972) empirically studied how people create and manipulate representations and improve intelligent information processing in tasks such as problem-solving.

A crucial innovation was the necessity to be selective in creating intelligent information processes. The world was all too large, and the size of exponentially growing search spaces easily surpassed the capacities of any machine. The way machines solved intellectual tasks was very different from the way people did it (Newell and Simon 1972; Saariluoma 1995). The difference between human intellectual information processing and that of machines was the human ability to concentrate on essential aspects of problems. Sifting out essential information from a mountain of inessential information requires effective selectivity.

The core concept thus became information selection. Intelligent machines must be able to select the right pieces of information from among irrelevant ones. To some degree, current intelligent artefacts can also do this. However, they still make very severe errors. Thus it makes sense to ask why exactly machines have difficulties in their information processing. Why do intelligent systems easily search for hundreds of thousands of irrelevant alternatives instead of concentrating on the sense-making ones? The answer to the problem of selection should be found in the basic conceptual structure of modern intelligent machines.

Intelligent machines are built on the concepts of mathematical or formal operations and sets. Although representations and processes are interpreted by people, intelligent information processing is grounded in formal concepts. However, there are no mathematical grounds to solve the problem of selection and determine which subset of a set of elements is relevant in a specific domain. It is always necessary to step outside of mathematics and formal thinking to define computational concepts that are relevant in a particular domain. This task can only be done by interpreting human minds. Differently from Turing’s (1950) assumption, on a general level machines do not think like people.

The problem is abstraction. The generality of Turing machines and other symbol-manipulating systems is reached by abstracting domain-specific information content and leaving it to people. As a consequence of formal abstraction, intelligent systems lose their ability to select relevant pieces of information from among all the possible information and disinformation. The problems of abstraction and relevance can often be solved in specific domains by good information systems work.

## Intelligence, information and ethics

Intelligent systems are able to process information in the way that people do. They have representations that are reasonably similar to those of humans, and they manipulate these representations in such a way that they are able to achieve at least as high a level of information processing as people do. Thus, the famous Turing (1950) test is actually a test of the goodness of performance of intelligent systems; it does not constitute proof that machines have the same general information processing capacity as people (Saariluoma and Rauterberg 2015).

An important aspect of the selectivity required in intelligent technologies is ethical information processing. Ethical rules and principles create guidelines on how people should act in different types of situations. These rules can be very elementary as in etiquette, for instance, at a Finnish coffee table people are supposed to take salty bread first, coffee bread next and cakes at the end. However, the guidelines can also be quite universal and complicated. For example, the golden rule (‘Treat others as you wish to be treated’) is applicable to many situations. The main issue here is that ethical and legal rules guide people’s actions, and thus they constitute information that can be used to select the lines of acting. Thus, ethics can be important in selective information processing.

## Weak and strong ethical AI

Improved computing speeds and the fast growth of data have made it possible to design technical artefacts with the ability to perform tasks that previously only people could carry out. Intelligent systems can execute tasks demanding intelligently selective information processing. In addition to fast routine processing of logical inferences, machines can decide between alternative courses of action. These systems can even learn selective classifications of their own, so that people are not able to forecast the information states that intelligent systems produce. Consequently, intelligent systems can selectively process information and choose between sense-making courses of action.

The capacity to engage in selective information processing makes it possible for modern AI and machine-based systems to compare values, which are associated with different information states on sense-making grounds. A chess-playing computer, for example, can select the best moves and courses of play out of millions of legal alternatives. Intelligent choices make machine actions intelligent. Similarly, machines can use ethical principles as heuristics to select between different actions.

Thus, machines can make ethically motivated decisions. They can, for example, classify people applying for health insurance as eligible or ineligible for a specific insurance program. Machines can also categorise people with different symptoms into those who will benefit from care and those whose pain is only prolonged by care. Thus, ethically motivated information processing is clearly possible.

However, ethical information processing is a complex problem. Ethical information processed by intelligent technologies can be divided into two main levels: (1) intelligent systems that can be given ethical rules and thus classify data based on ethical criteria and (2) systems that have the capacity to develop new and unforeseen ethical rules. The first type refers to intelligent machines that rely on human-implemented ethical heuristics created by humans or strongly ethical machines that can generate their own new ethical rules and principles. The examples described above demonstrate that ethical information processing systems in which people use ethically motivated classification criteria are easy to realise. Of course, the content of the rules is an issue of ethical, administrative, managerial and legal discourse, but there are no ethical problems associated with developing intelligent systems processing data. However, a more complex issue is whether one can also create ethical information processing systems that can derive ethical rules and principles by analysing data.

For the sake of clarity, the first type of information processing system can be called ethically weak intelligent or AI (WEAI). The second type, which can analyse data and generate new ethical principles, can be called strong ethical AI or intelligence. Weak ethical intelligence is not a problem, but strong ethical AI *is*. Ethically weak AI systems have been shown to be relatively easy to construct. They simply require describing an ethical classification criterion for processed data and information. However, constructing strong ethical AI is challenging as it requires making inferences from data to values or from how things *are* to how they *ought to be*. Strong ethical AI is limited by Hume’s guillotine, which is described in more detail in the next section. Although computational data science is vital today, the importance of Hume’s (1738/1972) work to AI ethics has not been addressed in AI ethics (Powers and Canascia 2020). Nevertheless, the differences between weak and strong AI makes Hume’s (1738/1972) core ethical thinking very relevant.

## Hume’s guillotine

David Hume (1738/1972, book 3/Sect. 1), the famous Scottish philosopher, analysed the ultimate grounds of human morals. His focus was on the relations of passions, what we would now refer to as emotions, and reasons, or cognitions, in current parlance. He argued that the function of morals was to excite, produce and prevent actions. In short, Hume maintained that morals play an important role in influencing what people do. The function of reason is to decide what is true and what is false, but it is impossible for reason to decide what is good or evil. Hume thus separated ‘what is’ from ‘what ought to be’ and claimed that it was impossible to derive the latter from the former.

Hume’s problem has several names such as Hume’s guillotine, Hume’s law and the ‘is/ought to’ problem. It is one of the traditionally most intriguing problems in ethics. It separates ethics from epistemology and appears to be somehow unintuitive. If a person learns that the excessive use of alcohol may easily lead to several kinds of illnesses, from liver problems to Korsakoff’s syndrome, should this mean that people should give up using alcohol?

Intelligent technologies are very practical for analysing large quantities of facts. However, it is unclear if large-scale information processing can be used to generate new ethical principles. Can our ethics be to some degree machine designed in the future? Could future society be AI governed so that intelligent machines make the laws based on the massive data people produce? Could politicians and administrative personnel do the same? In thinking about these kinds of somewhat sci-fi-type questions, Hume’s old aporia is obviously important.

The capacity to engage in selective information processing makes it possible for modern AI-based systems to compare the values of different information states on sense-making grounds. A chess-playing computer, for example, can identify the best sequences of moves among millions of legal alternatives (Saariluoma 1995). Intelligent choices make machine actions intelligent.

Facts are different from values. While facts can be true or false and thus are binary, values are not dichotomous. Something can be obliged, forbidden or allowed (v. Wright 1963). The problem of relations to binary facts in binary machines and multiple state values is important in designing ethical information systems and is conceptually important in designing ethically intelligent technologies. Hume’s guillotine appears to constitute an overwhelming obstacle to any kind of strong ethical AI.

However, Hume’s is-ought to problem seems somehow problematic and uneasy. If facts have no relation to values, how can people create ethical thinking? When the association between smoking and lung cancer was found in the early 1960s, medical doctors in particular gave up smoking. Clearly, facts had a meaningful connection to how people decided to act. For this reason, it is relevant to rethink Hume’s analysis and consider whether there is a link between factual information and values.

It seems reasonable to consider how people create their values in their real lives. One can put academic value discourse to one side and study the value formation in life itself. Eduard Westermarck (1906) promoted the idea of studying ethics in life and society. Instead of setting rules for marriage, he simply wanted to study how different people and cultures understand the rules of marrying. Sometimes people speak of Westermarck ethics. Thus, it makes sense to consider the cognitive and emotional processes involved in forming ethical principles and rules. This is a sensible question, and the relationships between passions and reason — or in our terms, emotions and cognitions — formed the foundations of Hume’s (1738/1972) thinking. Moreover, the relations between cognitions and emotions have been central to many analyses of ethics (MacIntyre 1967; Malik 2014).

## Emotions and cognitions

In the human mind, emotions are intimately linked with ethical information processing. This is natural, as emotions decide the value of things and actions to a person, and for this reason, emotions have had an important role in discussing the ethical mind. The emotion-based approach to ethics has been called emotivism.

Emotive ethics or emotivism serves as a good starting point for the present analysis of the ethical relevance of information processes. In human information processing, emotions represent an evolutionarily more basic system of thinking than cognition (Allman 2000). Emotional areas of the brain develop earlier than cognition, and especially higher-level cognition such as thinking.

Emotional ethics considers emotions to be fundamental components of ethical thinking. It was central to British empiricism. Smith (1976) and Hume (1738/1972), for example, recognised the importance of emotional processes or passions and sentiments. In the last century many important researchers such as Moore 1903) and Ayer (1936) have also supported emotivism in different forms.

In modern information processing concepts, the core property of emotions is valence. This concept refers to the negativity or positivity of emotions and feelings (Ekman, 1999, Frijda, 1988; Oatley et al., 2006; Thagard 2005). Feelings can be divided into opposite pairs such as pleasure or pain, good or bad, sorrow or joy and warmth or coldness. The opposite emotions represent examples of opposite valences. In ethical information processing, valence defines the goodness of actions and situations and thus is essential to deciding how positive or negative actions and respective situations are from an individual’s point of view. Valence also defines how much people want something.

Hume’s guillotine is closely linked to emotions or passions; it is ultimately based on the difference between emotions and reasons or cognition. Hume discussed the differences between reason and passion. He argued that the function of reason is to decide whether something is true or false. Emotions or passions with morals ‘produce or prevent actions’. The two mental faculties are separate in the sense that truth and falsehood, i.e. reason, cannot dictate emotions. Consequently, the difference between an act that is morally good or bad cannot be based solely on reason (Hume 1738/1972).

Human cognition refers to how people process information (Anderson 1993; Neisser 1967). Individuals take information from their environment, store and manipulate it. Thus cognition regulates their actions and, for example, provides information about the routes one could take to walk through a shop. Cognition encodes actions and thoughts that have led people to a particular situation and stores this information in long-term memory. Thus, cognition affords mental representations of actions and their consequences.

Several ethics frameworks have been based on cognitive rules. An example is Habermas (2018) and deontological ethics. Deontological or axiological ethics describe explicated norms of actions. The rules define ‘correct’ actions. The axiological norms are represented in the cognitive mind (Findlay 1970, Saariluoma and Rousi, 2020).

In real life, emotional processes are strictly linked to cognition. Emotional states activated by a particular situation require an individual’s cognitive understanding of the situation. If it is understood to be risky or threatening, the emotional states are constructed based on danger-related emotions, such as excitement, fear and courage. If the outcome of the cognitive analysis is positive, emotional states can be characterised by relaxation, happiness, humour and benevolence, for example. Before the situation-related emotional representation is constructed in the human mind, the cognitive content of representations must prevail (Frijda 1988, Power and Dalgleish 1996).

The psychological process that associates a situation’s cognitive and emotional representations is called appraisal. Appraisal is a core process in the psychology of emotions, which is often defined as the representation of an individual’s emotional significance, and the associated emotional value of cognitions and actions.

Cognitions generate cognitive aspects of ethical experiences in any situation. Emotions provide evaluative information about the value of given situations. Emotions also entail information about, for example, whether situations are pleasant or unpleasant, and good or bad. Thus, ethical experiences arise from both cognitions and emotions at the same time. The two systems encode different aspects of experiences and their respective components in mental representations. However, the cognitive and emotional dimensions of representation must both be active.

The problem with Hume’s (1738/1972) thinking is that he considered emotions and cognitions to be opposites, and separated them; for this reason he ended his guillotine. He argued that cognitions or reasons cannot dictate what is valuable: only emotions can do this. While this is true, there are no emotions without cognitions. Cognitive representations give rise to emotions, and thus one cannot have ethics without cognitions. The very basic question of Hume’s guillotine is mistaken since it is based on a psychologically incorrect conceptual discrimination.

People learn from experience to associate their actions with the situations these actions have led to. Based on these learned experiences, they encode rules of good conduct in interacting with technologies, including intelligent technologies (Thagard 2005). People learn to use them, which generates memory representations about the consequences of their actions and the reasons why particular types of actions should be avoided or pursued, i.e. are the actions or duties allowed or forbidden (Turing 1950). The representation of an action, its end situation and the emotional analysis of this situation can be called primary ethical representation.

Primary ethical representation entails a situation in the mind, including its emotional aspects. Since it is a unified whole, it entails information about experiences and subconscious information about any situation. Such representations can be stored in the memory as mental models or schemas. These learned long-term memory representations provide a basis for interpreting new situations (Saariluoma 1995).

## Social discourse

Ethics is social because people are social. Aristotle’s (1984, 1252a) idea of political humans articulates the social dimensions of the human mind. Human social actions organise people into an infinite number of types of social circles and contexts such as cultures, sports clubs, households, non-governmental organisations, states, schools, religious communities, campers, families, entrepreneurs and taxpayers. We define forms of life as social groups of all kinds that organise a participant’s actions around some system of rule-following actions (Habermas 1981, 2009).

Forms of life can thus be seen as organised systems of action in which individuals can participate; ethics is essential to devising rules for such groups. For instance, Catholics are supposed to participate in the ceremonies of the holy week. People’s actions follow the norms and traditions of the event. In families, most people strive to take care of their children and speak with them about the way people should live. Such structuring discourses belong to all forms of life.

Forms of life have rules, which can keep changing. A key mechanism of such changes is social discourse in its numerous forms (Habermas 1981). Social discourse entails communicating individual ethical rules and norms. People feel that something causes pain and identify the mechanism of action that led to those unpleasant feelings. The discourses in different contexts give people the ability to create common norms within society, which in turn shape the forms of life.

Social discourse can be as free as norm following. For example, discussions among friends are different from discourses in the meetings of enterprise executive boards. Today a portion of social discourse takes place in social media and thus is not formally normed. Even academic and political discourses on ethical and legal issues can be seen as aspects of social discourse. As a consequence of social discourses, various types of actions regulating rules normally emerge.

Ethical discourses initially help define how people should act in different forms of life. Social discourse creates formal and less formal regulatory rules, principles, norms and values. Societies are often regulated by laws. However, laws are outcomes of social discourse that are private as well as administrative or political. The forms of these discourses can vary from one society to another; democracies organise their discourses differently from oligarchies or dictatorships. Nevertheless, there are always groups of people that create new forms of life through thinking and discourse.

The social process of creating informal, tacit and formal regulatory rules and principles for different forms of life has been analysed in detail in discourse ethics (Habermas 2009). A key issue is that individual thoughts are submitted for social discourse in different forms. Ethical thoughts are often analysed by assessing the argumentation. If arguments are valid, it is possible to continue norming. However, if they are no longer valid, for instance, if historical changes have made many earlier rules outdated and thus in need of replacement, the arguments will also normally be replaced. However, history has shown that replacement processes are not always smooth. They can even be very violent.

Social discourses create socially shared rules and norms, which are continuously updated. Social attitudes keep changing, social experiences are communicated to other people, and the discourses converge into systems of tacit and explicit norms and values. As a whole, the system of emotional valences, social analysis of related actions and action types, as well as social discourse, give rise to ethical processes that create the values people follow in their lives.

Individuals’ primary ethical representations and schemas form the basis of social discourse. Through small and large, formal and informal discussions, people create their views about what are the most important and fundamental ethical experiences and respective rules. Discourse ethics has investigated this process (Habermas 2009).

In discourse ethics, representations are submitted to argumentative or foundational analysis. Each primary representation or ethical rule will be submitted to the foundational discourse. Any ethical rules that cannot be argumentatively supported will be rejected. The discourse itself has layers and sub-discourses. The main outcome is a system of ethical concepts, rules and principles. The unification of emotional, cognitive and social analysis can be called an ethical information process.

## Ethical information process

Real-life ethical information processes generate social ethics. This process creates values and norms. Thus, research on ethical processes should be an essential part of modern ethics, as it provides a realistic view of a society’s ethical thinking. Understanding the ethical information process also makes it possible to circumvent Hume’s guillotine. Hume’s (1738/1972) aporia seems to be the result of insufficient analysis of the relationship among people’s minds, ethical norms and actions.

Ethical information processes and their analyses represent a specific approach to the study of ethics, which can be supported by its importance in designing an ethical world. Instead of simply representing external academic norms related to the right kind of patient care, designers can work to understand how people are really taken care of, for example, in units for senior citizens, and what norms caretakers follow in their daily lives.

This type of empirical ethics is intimately connected to the analysis of ethical processes, but with an important difference. The former moves the focus from academic discussions to life as people live it, which leads to the tacit and explicit development of a society’s ethics, while the latter refers to the analysis of how norms are created. It is thus an empirical model of metaethical processes in real life.

Westermarck (1906) studied the norms and values of empirical ethics. The analysis of ethical information processes takes a slightly different form: it concentrates on the process of creating the social norms and ethical values people follow in their lives. The creation of values and following them as social processes are both important in research on ethical information processes. I refer to ethics based on the analysis of real-life value creation processes as ‘process ethics’ to distinguish it from more static approaches.

Value creation is important for design thinking. Design is a value generation process. If researchers understand the value creation process, they can improve it by providing empirical information on different aspects of the process. This shift from reflective to active involvement and influence is vital when designing ethical AI processes. Academic discussion is one example of a value generation process, but administrative, journalistic and law-making processes are equally important. The most important in an open society is nevertheless the discussions between citizens.

Ethical information processes can help circumvent Hume’s guillotine. Hume makes the fundamental (unsupported) assumption that emotions and cognitions are opposites in the human mind, and for that reason cannot affect emotions. However, there is no support for such a conceptual differentiation in modern research on the mind (Power and Dagleish 1996). One cannot conclude that the two concepts are opposites based on the fact they are different. It is possible that they complement each other.

Emotions and cognitions jointly regulate human actions. They have different functions, and both are necessary. Emotions determine the goodness or badness of actions and attribute personal meaning to individuals, while cognitions analyse actions and consequential situations. Thus, the two faculties together can construct ethical experiences and primary ethical schemas. Social discourse turns these primary ethical experiences and schemas into socially agreed rules and even laws. In this way they perform functions within relevant forms of life. Thus, Hume’s guillotine is a pseudo problem.

## Discussion

Ethical and moral norms often begin with observations. In the case of protecting people from the coronavirus, numerous ethical and legal norms have been generated in a very short period. Governments have closed schools, borders and restaurants. They have implemented numerous limitations on everyday human life. These regulations are ethical norms that have been grounded in factual experience from around the world, especially from China, where the problem became large scale.

Thus, facts formed the basis of values in ethical information processes. Emotional analyses reveal the consequences of corona-induced illnesses and feelings. Cognitions were used to select correct and effective ways to respond to the problems. The responses were ethical norms turned into laws through administrative and parliamentary discourses. This is a good example of how ethical information processes operate. Hume, 1972) guillotine does not prevent individuals from using cognitive information to derive values if emotions and cognition are not separated and juxtaposed.

A glimpse at ethical processes provides no fundamental obstacle to using intelligent technologies to develop new norms. It is possible to classify situations into ethically and emotionally pleasant or unpleasant. Problem-solving methodologies can also be used to generate alternative situations. Thus, it may also be possible to generate solutions for action problems that have ethical dimensions. The main problem is that people have different types of values; thus it is hard to see that machines can solve what can be characterised as human discursive positions.

The fall of Hume’s guillotine paves the way for the development of strong ethical AI systems. Such systems can be used to analyse data and visualise its connections to actions. Machines can also help determine whether the resulting situations are emotionally pleasant or unpleasant. By combining the facts with emotional valence information concerning particular situations, machines can discover new primary values for social discourse. They can construct primary ethical schemas and thus develop stronger ethical AI. Human social discourse would be required to decide whether these new primary schemas are valid.

The analysis presented here suggests that there are two poles in ethical information processing, which can be called weak and strong ethical AI. Ethically weak AI systems can apply given ethical rules in certain situations. They can recognise critical features in situations and choose their actions on this basis. In such cases, ethics are just a human-implanted feature in a recognition action system.

Ethically strong AI systems can generate ethical norms for people. They should be able to analyse situations, for instance, in terms of their possible pleasantness or unpleasantness. They could also generate alternative courses of action and evaluate their potential consequences. The hard part would be deciding what human ethics should be and bypassing human discourse. Thus, people can use ethical AI programs to help them generate their ethical rule systems: machines could warn about risks and suggest an alternative course. Thus it seems the final decision should be done by human ethical analyses and discourses.

Partially strong AI is already a reality. Statistical analysis, forecasting schemas and network communication currently provide tools for active human ethical regulation. The step to next-generation AI supporting practical ethical work is not nearly as dramatic as it appears to be. AI can help people in many ways in their work to generate new ethics during the transition period.

People will have to overcome several types of crises in the near future in addition to the coronavirus. Population crises, pollution crises, climate change, economic transformation and collapses and new kinds of illnesses may be on the horizon. It may be that people can no longer continue to live in the same way they have in the past. In innovating out of the abyss, the effective use of computing has an important role.

It is possible to code the ethical values of situations in different ways and to teach machines to avoid painful states and to strive for rewarding situations. This was already proposed by Turing (1948). The purpose of the argument presented here is only to show that there are no conceptual barriers to the ethical analysis by means of stronger artificial intelligence. Therefore, it is essential to work with ethical AI and AI ethics. Hume’s guillotine does not represent untwisted conceptual obstacle.

## References

[CR1] Allman, J (2000) Evolving brains. Scientific American / Freeman New York.

[CR2] Anderson JR (1993). Rules of the mind.

[CR3] Aristotle (1984) Politics. In: Barns J (ed.) Complete works of Aristotle. Princeton University Press, Princeton, NJ

[CR4] Ayer A (1936). Language, logic and truth.

[CR5] Bernal JD (1969). Science in history.

[CR6] Bryson, J. (2020) The artificial intelligence of the ethics of artificial intelligence. In MD, Dubber, F. Pasquale, S. Das (eds), The Oxford handbook of ethics of AI. Oxford University Press, Oxford

[CR7] Chandler D (2007). Semiotics.

[CR8] Dignum V (2019). Responsible artificial intelligence.

[CR9] Ekman P (1999) Basic emotions. In Dalgleish, T, Power M (eds), Handbook of Cognition and Emotion. Wiley, Chichester

[CR10] Findlay J (1970). Axiological ethics.

[CR11] Floridi L (2011). The philosophy of information.

[CR12] Ford M (2015). Rise of the robots.

[CR13] Frijda NH (1988). The laws of emotion. Am Psychol.

[CR14] Fukuda K (2020). Science, technology and innovation ecosystem transformation toward society 5.0. Int J Prod Econ.

[CR15] Gungel D (2012). The machine question.

[CR16] Habermas J (1981) Theorie des kommunikativen Handelns 1–2. [Theory of communicative action]. Suhrkamp, Frankfurt am Main

[CR17] Habermas J (2018). Diskursethik [Discourse ethics].

[CR18] Harnad S (1990). The symbol grounding problem. Physica, D.

[CR19] Headrick D (2009). Technology and world history.

[CR20] Hintikka J, Sandu,G (1997). Game theoretical semantics. In: van Bentheim, J, ter Meulen A (eds.), Handbook of logic and language. Elsevier, Amsterdam.

[CR21] Horne, A. (1962) Verdun 1916. (The price of glory) WSOY, Porvoo

[CR22] Hume D (1972/ orig. 1738) A Treatise of Human Nature. Dent, London

[CR23] Kåhre J (2002). The mathematical theory of information.

[CR24] Kant, I (1781/1976) Kritik der reinen Vernunft. [The critique of pure reason]. Felix Meiner, Hamburg

[CR25] Leikas J, Koivisto R, Gotscheva N (2019). Ethical framework for designing autonomous intelligent systems. Journal of Open Innovation.

[CR26] Lyons J (1977). Semantics 1–2.

[CR27] MacIntyre A (1967). A short history of ethics.

[CR28] Malik K (2014). The quest for moral compass: the global history of ethics.

[CR29] Moore G (1903). Principia ethica.

[CR30] Mueller, V (2020) Ethics of artificial intelligence and robotics. In Zalta E (ed), Stanford encyclopaedia of philosophy, Stanford.

[CR31] Neisser U (1967). Cognitive Psychology.

[CR32] Newell A, Simon HA (1972). Human problem solving.

[CR33] Newell A, Shaw J, Simon H (1958). The elements of a theory of human problem solving. Psychological Review.

[CR34] Nierenburgh S, Raskin V (2004). Ontological semantics.

[CR35] Oatley K, Keltner D, Jenkins JM (2006). Understanding emotions.

[CR36] Ogden C, Richards I (1923). The meaning of meaning.

[CR37] Petzold C (2008). The annotated Turing.

[CR38] Power M, Dalgleish T (1997). Cognition and emotion.

[CR39] Powers, T, Canascia JG (2020) The ethics of the ethics of AI. In MD, Dubber, F. Pasquale, S. Das (eds), The Oxford handbook of ethics of AI. Oxford University Press, Oxford.

[CR40] Saariluoma P (1995). Chess players’ thinking.

[CR41] Saariluoma, P (2000) Kognitiotieteellinen semantiikka. [Cognitive science and semantics]. In: Airola, A, Koskinen H & Mustonen, V, (eds.) Merkillinen merkitys [Strange meaning]. (pp. 44–68). Helsinki: Gaudeamus.

[CR42] Saariluoma, P (2012) Muotokokemusten sisältöjen kognitiotieteellisestä analyysistä. [The cognitive analysis of form experiences]. In: Kähkönen, S, Lähdesmäki, T (eds.) Tieteidenvälisyys ja rajanylitykset taidehistoriassa. [Interdisciplinary research in art history] (pp. 49–56). The Finnish society of art history, Helsinki

[CR43] Saariluoma P, Rousi R (2015). Symbolic interactions: towards a cognitive scientific theory of meaning in human technology interaction. J Adv Humanit.

[CR44] Saariluoma P, Rauterberg M (2015) Turing test does not work in theory but in practice. In: Arabnia, HR, Fuente, D, Dziegiel R, Kozerenko E, LaMonica H, Liuzzi A, Waskiewicz T, (eds.), ICAI 15: Proceedings of the 17th International Conference on Artificial Intelligence (pp. 433–437). WORLDCOMP. Retrieved from http://worldcomp-proceedings.com/proc/p2015/ICA3164.pdf. Accessed 1 9 2019

[CR45] Saariluoma P, Cañas J, Leikas J (2016). Designing for life.

[CR46] Saariluoma, P Rousi, R. (2020) Emotions in technoethics. In Rousi, R, Leikas, J Saariluoma, P (eds.) Emotions in technology design: from experience to ethics. Springer: Cham

[CR47] Saussure F (1919/1983) Course in general linguistics. Duckworth, Guilford

[CR48] Schön L (2013) Maailman taloushistoria [The economic history of the world]. Vastapaino, Tampere

[CR49] Searle J (1992). The Rediscovery of mind.

[CR50] Shannon C (1948) A mathematical theory of communication. Bell Syst Tech J 27(379–423):623–656

[CR51] Shannon C, Weaver W (1949). The mathematical theory of communication.

[CR52] Skinner B (1957). Verbal behaviour.

[CR53] Smith A (1976). A theory of moral sentiments.

[CR54] Thagard P (2005). Mind.

[CR55] Tegmark M (2017) Life 3.0. Penguin Books, Harmondsworth

[CR56] Turing AM (1937). On computable numbers, with an application to the entscheidungsproblem. Proc London Math Soc.

[CR57] Turing AM (1950). Computing machinery and intelligence. Mind.

[CR58] Turing AM (1948) Intelligent machinery. In Copeland, J. (ed.), The essential Turing. Claredon Press: Oxford (2004).

[CR59] v. Wright G (1963) Norm and action. Routledge and Kegan Paul, London

[CR60] Westermarck E (1926). The origin and development of moral ideas.

[CR61] Wiener N (1948). Cybernetics.

[CR62] Wittgenstein L (1953). Philosophical investigations.

[CR63] Yang G (2018). The grand challenges of science robotics. Sci Robot.

